# Potential role of cardiopulmonary exercise testing in evaluating functional improvement after transcatheter edge-to-edge tricuspid valve repair: a case report

**DOI:** 10.1093/ehjcr/ytae102

**Published:** 2024-03-05

**Authors:** Luca Cumitini, Ailia Giubertoni, Marco Giovanni Mennuni, Anna Degiovanni, Giuseppe Patti

**Affiliations:** Department of Translational Medicine, University of Eastern Piedmont, Via Solaroli 17, Novara 28100, Italy; Department of Thoracic and Cardiovascular Diseases, Maggiore della Carità Hospital, Via Mazzini 18, Novara 28100, Italy; Department of Thoracic and Cardiovascular Diseases, Maggiore della Carità Hospital, Via Mazzini 18, Novara 28100, Italy; Department of Thoracic and Cardiovascular Diseases, Maggiore della Carità Hospital, Via Mazzini 18, Novara 28100, Italy; Department of Thoracic and Cardiovascular Diseases, Maggiore della Carità Hospital, Via Mazzini 18, Novara 28100, Italy; Department of Translational Medicine, University of Eastern Piedmont, Via Solaroli 17, Novara 28100, Italy; Department of Thoracic and Cardiovascular Diseases, Maggiore della Carità Hospital, Via Mazzini 18, Novara 28100, Italy

**Keywords:** Tricuspid regurgitation, Transcatheter edge-to-edge repair, Cardiopulmonary exercise testing, Echocardiography, Case report

## Abstract

**Background:**

Tricuspid regurgitation (TR) is common and severe or greater TR is linked to poor prognosis. Treatment of TR with transcatheter edge-to-edge repair has emerged as a safe and potentially effective therapy in these patients. However, the impact of transcatheter tricuspid repair on functional capacity remains to be elucidated.

**Case summary:**

We describe the case of a 77-year-old woman complaining of heart failure symptoms, undergoing transcatheter edge-to-edge valve repair for severe TR with the PASCAL Ace® device. One month later, cardiopulmonary exercise testing (CPET) showed significant improvement in peak O_2_ uptake and O_2_ pulse compared with the test performed before the procedure.

**Discussion:**

A positive impact of novel transcatheter edge-to-edge valve repair on symptoms and quality of life in patients with severe or greater TR at prohibitive surgical risk has recently emerged. The presence of severe TR has prognostic relevance, and novel percutaneous tricuspid valve repair systems have emerged in the last few years. Cardiopulmonary exercise testing is an established tool to assess functional capacity and prognosis in heart failure patient. Detecting functional capacity improvement after transcatheter edge-to-edge repair for severe TR can be challenging, and CPET may arise as a promising tool to help these purposes.

**Learning points**

- Techniques of transcatheter edge-to-edge repair for severe tricuspid regurgitation in patients at prohibitive surgical risk are rapidly increasing.
- Cardiopulmonary exercise testing may represent a promising tool for detecting functional capacity improvement after transcatheter edge-to-edge tricuspid valve repair.

## Introduction

Tricuspid regurgitation (TR) is common, having a prevalence of >60% in the Western population.^1^ Severe or greater TR is linked to a poor prognosis with an estimated 5-year survival rate of 30% compared with individuals without relevant TR.^1^ Recent acknowledgement that the presence of TR has independent prognostic implications for the subsequent clinical outcome has refocused attention on treatment options.^2^ Medical treatment does not impact on survival in this setting of patients. Surgical treatment of isolated TR is often challenging, due to concomitant patient-related factors increasing the post-operative risk, including right ventricular dysfunction or hepatorenal failure secondary to chronic venous hypertension.^2^ Transcatheter edge-to-edge repair (TEER) has emerged as a safe and potentially effective treatment for patients with severe TR.^3^ However, the subsequent impact of this transcatheter tricuspid repair on functional capacity remains to be established.

## Summary figure

**Figure:**
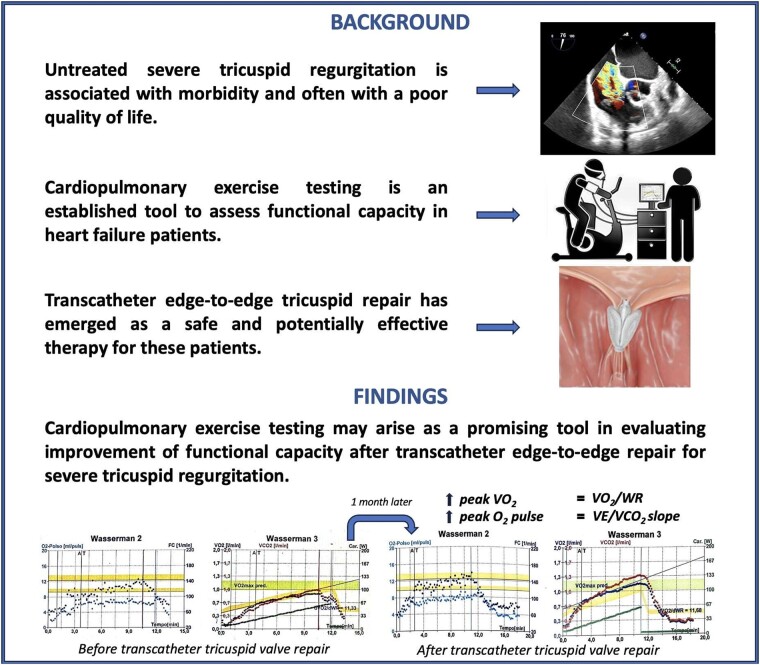


## Case presentation

**Figure 1:**
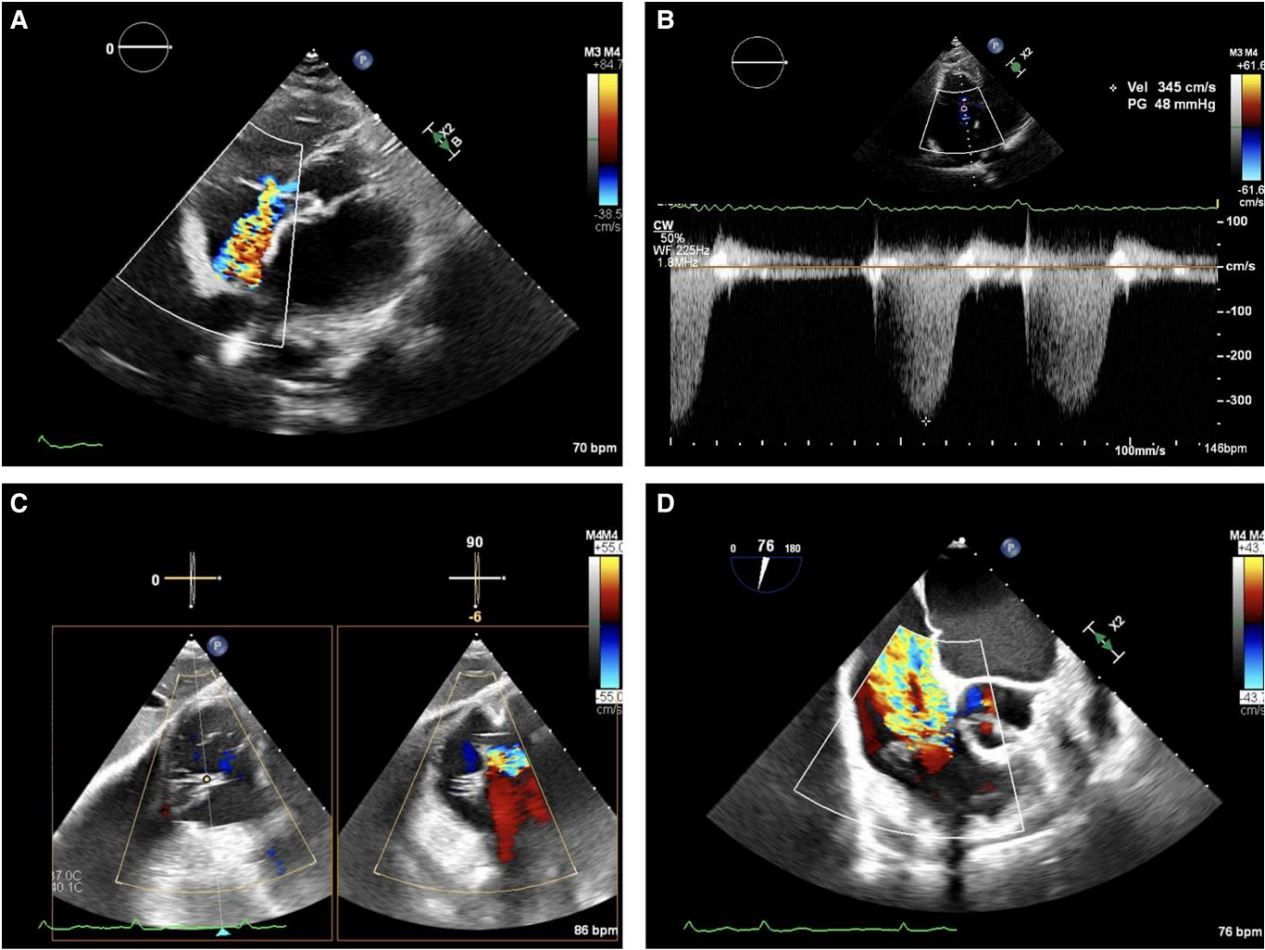
Transthoracic and transoesophageal echocardiography on admission. (*A* and *B*) Transthoracic echocardiography by apical five-chamber view showed severe tricuspid regurgitation, with tricuspid regurgitation pressure gradient of 48 mmHg. Transgastric (*C*) and mid-oesophageal (*D*) short-axis view at transoesophageal echocardiography confirmed severe tricuspid regurgitation.

A 77-year-old woman was admitted to our institution complaining of progressive leg swelling and worsening exertional dyspnoea. Her past history was characterized by arterial hypertension, cigarette smoking, chronic obstructive pulmonary disease, permanent atrial fibrillation (AF) from 9 years, and previous hospitalizations for heart failure episodes. A diagnosis of TR was made 3 years before admission. She was independent with activities of daily life pre-admission. At the time of admission, physical examination showed lower limb oedema, jugular vein distention, and 2/6 pansystolic murmur heard loudest at the left lower sternal edge, suggestive of TR. Body temperature was 36.8°C, heart rate was 102 b.p.m., saturation on room air oxygen was 96%, and blood pressure was 132/85 mmHg. Atrial fibrillation rhythm was present at electrocardiography, and pulmonary congestion was revealed by clinical visit and confirmed by chest radiography. Blood tests showed normal renal and liver function. Brain natriuretic peptide (BNP) was 580 pg/mL (normal value < 60 pg/mL). Preserved left ventricular ejection fraction (55%), borderline parameters of filling pressures in the left ventricle (*E*/*e*ʹ average 9), augmented left ventricular mass index (142 g/m^2^), TR velocity at rest (3.4 m/s), normal right ventricular function [tricuspid annular plane systolic excursion (TAPSE) of 22 mm, *S*ʹ right ventricular wave of 10 cm/s, and fractional area change of 46%] and size (basal right ventricular diameter of 37 mm), and severe left and right atrium enlargement (left atrium volume index of 80 mL/m^2^ and right atrium area of 43 cm^2^ in apical four-chamber view) were demonstrated at transthoracic echocardiography (TTE) examination. Moderate mitral valve regurgitation and severe TR were detected at colour flow Doppler imaging, with a TR pressure gradient of 48 mmHg. A mild dilatation (23 mm) with preserved collapsibility was observed regarding in the inferior vena cava evaluation. Transoesophageal echocardiography (TEE) confirmed severe TR [vena contracta 3D of 60 mm^2^, vena contracta in *x*-plane view of 0.9 × 0.20 cm, and effective regurgitant orifice area (EROA) of 51 mm^2^], with prolapse of the septal leaflet, rupture of a septal cord, and dilatation of the tricuspid annulus (anteroposterior diameter of 44 mm) (*Figure 1*; see Supplementary material online, *Videos S1* and *S2*). Infusion of diuretic drugs (furosemide and canrenone) was early started, with the initial improvement of symptoms and signs of heart failure. For assessing patient’s functional capacity, as well as cardiopulmonary and metabolic changes, a cardiopulmonary exercise testing (CPET) was performed using a cycle ergometer and lasting 10 min. A continuous 5 W incremental ramp protocol minute-by-minute (basal ramp of 10 W) was used, reaching maximal metabolic effort (respiratory exchange ratio of 1.10). Electrocardiography, blood pressure, and peripheral oxygen saturation were documented before, during, and after exercise. An arterial blood gas test was obtained during the resting phase and at maximum effort. Cardiopulmonary exercise testing showed a mildly reduced functional capacity, with peak oxygen uptake (VO_2_) of 12 mL/kg/min (82% predicted), reduced peak O_2_ pulse (6.2 mL/beat, 66% predicted), and normal peak oxygen uptake to work rate slope (VO_2_/WR, 11 mL/min/W). A mild pulmonary vascular impairment, with minute ventilation to carbon dioxide production slope (VE/VCO_2_) of 31.8, and normal ventilatory parameters, with a breath reserve of 48%, were detected. A normal anaerobic threshold was found. Right heart catheterization showed combined pre- and post-capillary pulmonary hypertension, with mean pulmonary arterial pressure of 35 mmHg, pulmonary vascular resistance of 5 wood units, and pulmonary arterial wedge pressure of 16 mmHg. No difference in pulmonary and wedge pressure was observed after fluid challenge test. A reduced cardiac index of 2.1 L/min/m^2^ was found. No oxygen saturation gap between the pulmonary artery and right atrium was present.

**Figure 2:**
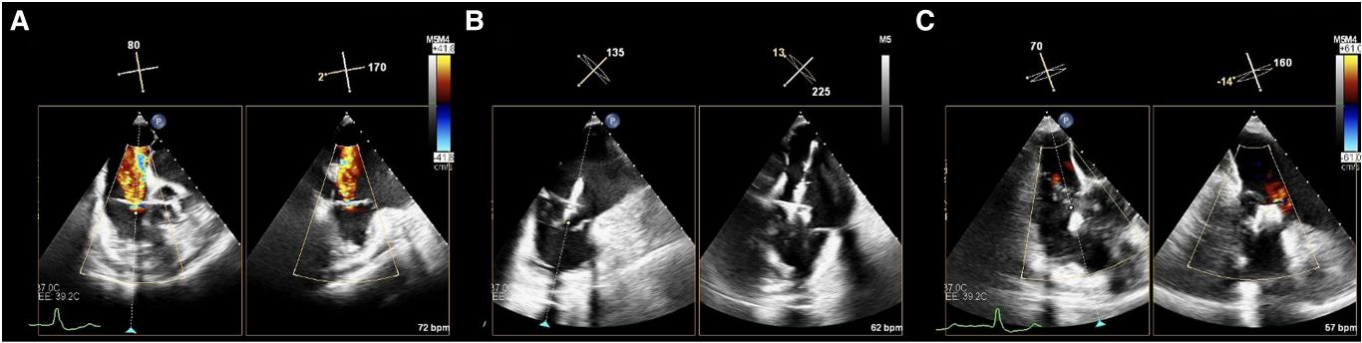
Periprocedural *x*-plane mode transoesophageal echocardiography. (*A*) Tricuspid regurgitation before the procedure, (*B*) during the procedure, and (*C*) after the procedure.

**Figure 3:**
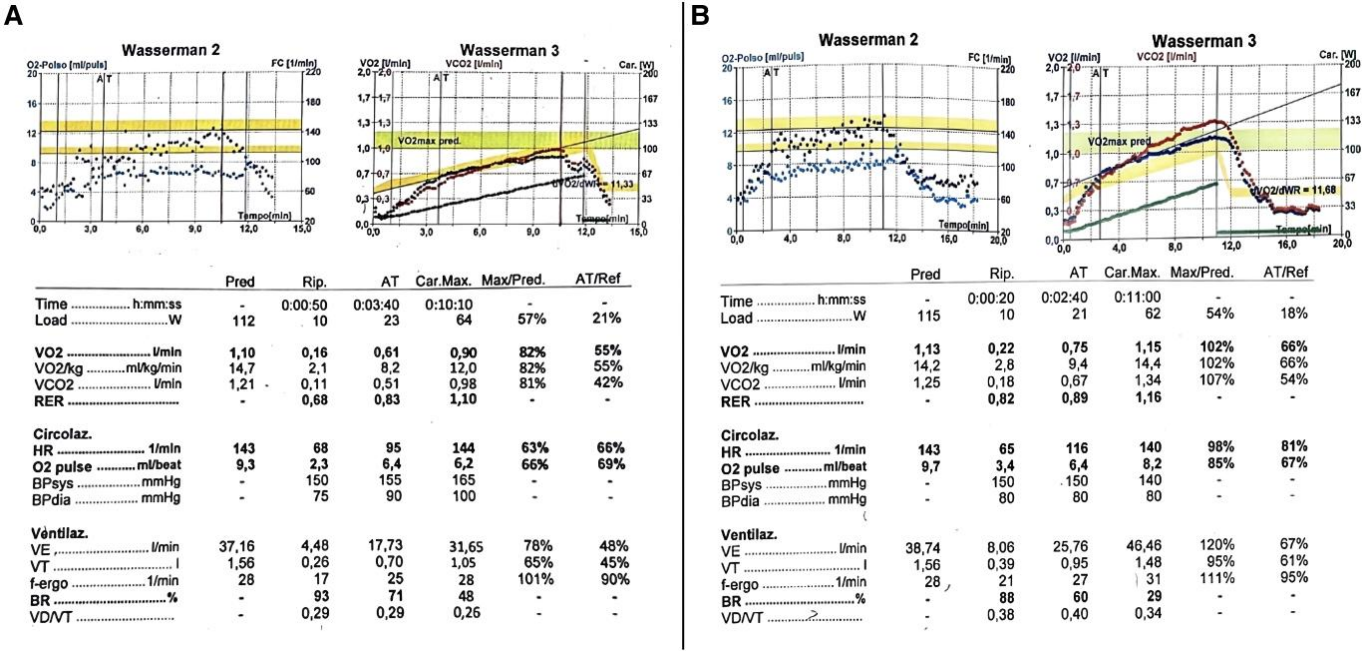
Cardiopulmonary exercise testing at diagnosis (left-sided panel, *A*) and at re-assessment after the intervention (right-sided panel, *B*). Peak oxygen uptake (14.4 mL/kg/min, 102% of predicted vs. 12 mL/kg/min, 82% of predicted) and peak O_2_ pulse (8.2 mL/beat, 85% of predicted vs. 6.2 mL/beat, 66% of predicted) were higher at reassessment. Only Wasserman panels 3 and 4 are shown.

Given the aforementioned clinical and imaging findings, a diagnosis of severe TR was made. Tricuspid regurgitation was both primary (prolapse of the septal leaflet) and functional, due to AF-related right atrium enlargement and tricuspid annulus dilatation. Because of subsequent, progressive right-side heart failure despite diuretic agents (furosemide and canrenone) and given the high surgical risk (TRI-SCORE 5 of 12; 14% predicted in-hospital mortality), our heart team decided to perform tricuspid TEER. This intervention was considered indicated also because pulmonary hypertension was mild, with a pre-capillary component due to chronic obstructive pulmonary disease and a post-capillary component due to mitral regurgitation and impairment of left ventricular diastolic function. Transcatheter edge-to-edge repair was done using the PASCAL Ace® system (Edwards Lifesciences, Irvine, CA, USA). The intervention was performed under general anaesthesia and TEE plus fluoroscopic guidance. The device was advanced using a 22-French guiding catheter via the right femoral vein to access the right atrium and implanted between anterior and septal leaflets. A reduction of TR from severe to mild was achieved after the procedure (*Figure 2*; see Supplementary material online, *Video S3*). The intervention was free from complications. The patient was discharged after 3 days, without symptoms and in good haemodynamic status. A complete re-evaluation was scheduled at 1 month after the procedure, when the patient reported marked subjective symptoms improvement, no longer complaining of dyspnoea or leg swelling. Repeat TTE demonstrated normal right ventricular function (TAPSE of 23 mm) and size (37 mm), with mild TR (vena contracta in *x*-plane view of 0.23 × 0.10 cm; EROA of 8 mm^2^; see Supplementary material online, *Video S4*) and TR pressure gradient of 28 mmHg. At blood tests, BNP was reduced to 80 pg/mL. Cardiopulmonary exercise testing was re-performed using the same pre-procedure ramp protocol. It was maximal for metabolic effort (respiratory exchange ratio of 1.16) and showed normalization of functional capacity, with a peak VO_2_ of 14.4 mL/kg/min (102% predicted) and peak O_2_ pulse of 8.2 mL/beat (85% predicted). VO_2_/WR slope, anaerobic threshold, and VE/VCO_2_ slope were approximately unchanged (*Figure 3*).

## Discussion

A positive impact of novel TEER on symptoms and quality of life in patients with severe or greater TR at prohibitive surgical risk has recently emerged.^2–4^ In particular, studies demonstrated a significant reduction of TR being associated with symptom improvement.^2^ Cardiopulmonary exercise testing is a unique tool able to objectively and simultaneously evaluate cardiopulmonary and metabolic changes and to assess functional capacity. In contrast to exercise electrocardiography, the direct non-invasive determination of minute ventilation, heart rate, and expired gases analysis at rest and during exercise in the CPET provides accurate and reproducible data on the interaction between ventilation, gas exchange, and cardiovascular/musculoskeletal function. Notably, CPET may predict outcome in patients with heart failure and other pathological conditions.^5^ However, the impact of transcatheter repair for TR on objective improvement of functional capacity, as assessed by CPET, to date remains to be established. Our report presents a case of evaluation by CPET in a patient treated with TEER for severe primary and secondary TR (predominantly functional, due to AF-related right atrial dilatation).

An increase in cardiopulmonary exercise capacity following transcatheter tricuspid repair was reported only by Volz *et al*.^6^ in a retrospective study on a small number of patients. In agreement with the results of this investigation, we observed after the procedure a significant improvement in peak VO_2_ and O_2_ pulse, without any change in VE/VCO_2_ slope. However, as compared with Volz’s study, in our patient, the pre-procedural peak VO_2_ was higher, whereas VE/VCO_2_ slope was almost normal, suggesting a mild secondary vascular pulmonary involvement. These features highlight how, in our case, TEER was not performed at the stage of advanced heart failure, where, as observed in Volz’s cohort, it might be futile and can carry a limited prognostic improvement. Nevertheless, in the latest European Society of Cardiology guidelines for the management of heart valvular disease,^4^ optimal timing for tricuspid TEER is not still suggested. We demonstrated an even earlier functional capacity improvement by CPET (at 1 month vs. 3 months in Volz’s investigation).

Prognostic implications of peak VO_2_ change in patients with chronic heart failure are well established. In particular, every 6% increase in peak VO_2_ has been associated with 8% lower risk of cardiovascular death or hospitalizations for heart failure.^7^ Thus, a peak VO_2_ improvement of 20%, as observed in our patient, can be potentially related to a clinical improvement with subsequent prognostic impact. Likewise, a lower peak O_2_ pulse has been correlated with major clinical events, such as cardiovascular death and urgent transplantations.^8^ Therefore, a peak O_2_ pulse improvement of 19%, as demonstrated in our case report, may be potentially relevant for survival benefit.

## Conclusion

In conclusion, the presence of severe TR has prognostic relevance, and novel percutaneous tricuspid valve repair systems have emerged in the last few years. Detecting functional capacity improvement after TEER for severe TR can be clinically relevant, and CPET may arise as a promising tool to help these purposes.

## Supplementary Material

ytae102_Supplementary_Data

## Data Availability

The data underlying this article are available in the article and in its online Supplementary material.

## Lead author biography

Luca Cumitini was born in Turin, Italy, in 1993. He graduated as a medical doctor in the School of Medicine of Turin University in 2019. He is currently a cardiology resident in the Department of Thoracic and Cardiovascular Diseases of the University of Eastern Piedmont, Novara, Italy. He has a special interest in heart failure and intensive cardiac care.
